# Reply to Pisan et al.: Pathogenicity of inherited TRAF7 mutations in congenital heart disease

**DOI:** 10.1073/pnas.2319578121

**Published:** 2024-03-11

**Authors:** Ketu Mishra-Gorur, Tanyeri Barak, Leon D. Kaulen, Octavian Henegariu, Sheng Chih Jin, Stephanie Marie Aguilera, Ezgi Yalbir, Gizem Goles, Sayoko Nishimura, Danielle Miyagishima, Lydia Djenoune, Selin Altinok, Devendra K. Rai, Stephen Viviano, Andrew Prendergast, Cynthia Zerillo, Kent Ozcan, Burcin Baran, Leman Sencar, Nukte Goc, Yanki Yarman, A. Gulhan Ercan-encicek, Kaya Bilguvar, Richard P. Lifton, Jennifer Moliterno, Angeliki Louvi, Shiaulou Yuan, Engin Deniz, Martina Brueckner, Murat Gunel

**Affiliations:** ^a^Department of Neurosurgery, Yale School of Medicine, New Haven, CT 06510; ^b^Department of Genetics, Yale School of Medicine, New Haven, CT 06510; ^c^Cardiovascular Research Center, Cardiology Division, Department of Medicine, Massachusetts General Hospital and Harvard Medical School, Boston, MA 02129; ^d^Department of Pediatrics, Yale School of Medicine, New Haven, CT 06510; ^e^Yale Cardiovascular Research Center, Department of Internal Medicine, Section of Cardiology, Yale School of Medicine, New Haven, CT 06510; ^f^Laboratory of Human Genetics and Genomics, The Rockefeller University, New York, NY 10065; ^g^Yale Program in Brain Tumor Research, Yale School of Medicine, New Haven, CT 06510; ^h^Department of Neuroscience, Yale School of Medicine, New Haven, CT 06510

We are in receipt of Pisan et al.’s letter (1). Our manuscript (2) reported the association of p.Val142Met, p.Val442Met, and c.1998+2T>G TRAF7 variants with congenital heart disease (CHD) based on several levels of data:

Experimentally, overexpression of p.Val442Met consistently phenocopies TRAF7 knockdown in *Xenopus* and *zebrafish*. Specifically, both impact neural crest development with disruption of *Sox10* and *Twist* expression; cause cardiac and craniofacial defects; disrupt ciliogenesis; and biochemically p.Val442Met, like meningioma-associated mutations, interferes with the TRAF7-CYLD and -IFT57 interactions

Several in silico tools strongly support the pathogenicity of these variants. Both missense mutations are predicted pathogenic by AlphaMissense and MetaSVM, with high REVEL scores, while 1998+2T>G is predicted to disrupt normal splicing. Both have low minor allele frequencies (7.72e−6 and 1.59e−6, respectively) in the general population, while the specific 1998+2T>G variant is not found in gnomAD-version4 (five times larger than version2).

Mutations outside the TRAF7 WD40 region are pathogenic in syndromic cases (3, 4) and cancer (5) including meningiomas, where they co-occur with KLF4/PIK3K-pathway mutations (6). Notably, cbioportal shows a wide distribution of *TRAF7* mutations (including p.Val142 and p.Val442) in a large cohort of cancer patients (https://bit.ly/3svbC5z).

A low pLI score alone is not predictive of non-pathogenic status of genes/variants. Indeed, several OMIM genes with low pLI scores (<0.1) cause autosomal dominant diseases (e.g., *COL4A2* 7). Further, heterozygous *Traf7* loss causes phenotypes (eye abnormalities and ECG changes, International Mouse Phenotyping Consortium), supporting haploinsufficiency of *Traf7*.

The absence of PDA in our cases is likely due partly to the documented exclusion of probands with isolated PDA from the Pediatric Cardiac Genomics Consortium (PCGC) cohort, which focuses on severe CHD. Nonetheless, our three probands show phenotypic overlap with previous reports (atrial septal defects, pulmonary atresia, and ventricular hypoplasia) (2, 4), including polydactyly (8, 9). Minimal phenotyping of parents in PCGC limits any further phenotypic correlations, including possible incomplete penetrance (2).

Gordon et al. themselves report only one child presenting the CHD phenotype (3) in a family of a mother with dizygotic twins carrying p.Phe617Leu mutation. Similarly, a p.Val646Ile mutation presents differently in a mother and son, with only the mother displaying polydactyly and dysmorphic facial features (9). Moreover, phenotypes of probands with identical missense mutation documented substantial variability of cardiac phenotypes (4), supporting variable expressivity of *TRAF7* mutations.

Finally, the absence of heart looping defects in *Traf7* knock-out mice (10) is likely due to different approaches (knockout versus knockdown), phenotypic discordance between animal models, and/or limitations of mouse models in recapitulating human CHD (11). Importantly, *Traf7* knock-out mouse study (11) supports our finding (2) of TRAF7’s role in cardiac development.

In our view, prior published results should not be assumed to be comprehensive. Our peer-reviewed paper, citing only the original study (4) with *TRAF7* germline mutations, reported significant genetic and biochemical research beyond the clinical genetics correlating TRAF7-related ciliary defects in meningiomas and CHD and broadened the disease spectrum. Pisan et al.’s unpublished mutations and phenotypes were not available to us, precluding any commentary. We believe that additional experimental studies will further reveal pathophysiological mechanisms underlying *TRAF7* mutations and determine the full spectrum of associated phenotypes.
